# Predicted effects of landscape change, sea level rise, and habitat management on the extirpation risk of the Hawaiian common gallinule (*Gallinula galeata sandvicensis*) on the island of O‘ahu

**DOI:** 10.7717/peerj.4990

**Published:** 2018-06-22

**Authors:** Charles B. van Rees, J. Michael Reed

**Affiliations:** Department of Biology, Tufts University, Medford, MA, United States of America

**Keywords:** Climate change, Connectivity, Habitat fragmentation, Hawaii, Moorhen, Population viability analysis, Wetland, Vortex, Rallidae, Island bird

## Abstract

We conducted a spatially explicit, stochastic, individually based population viability analysis for the Hawaiian common gallinule (*Gallinula galeata sandvicensis)*, an endangered subspecies of waterbird endemic to fragmented coastal wetlands in Hawai‘i. This subspecies persists on two islands, with no apparent movement between them. We assessed extirpation risk for birds on O‘ahu, where the resident gallinule population is made up of several fragmented subpopulations. Data on genetic differentiation were used to delineate subpopulations and estimate dispersal rates between them. We used sensitivity analyses to gauge the impact of current uncertainty of vital rate parameters on population projections, to ascertain the relative importance of gallinule vital rates to population persistence, and to compare the efficacy of potential management strategies. We used available sea level rise projections to examine the relative vulnerability of O‘ahu’s gallinule population to habitat loss arising from this threat. Our model predicted persistence of the island’s gallinule population at 160 years (∼40 generations), but with high probabilities of extirpation for small subpopulations. Sensitivity analyses highlighted the importance of juvenile and adult mortality to population persistence in Hawaiian gallinules, justifying current predator control efforts and suggesting the need for additional research on chick and fledgling survival. Subpopulation connectivity from dispersal had little effect on the persistence of the island-wide population, but strong effects on the persistence of smaller subpopulations. Our model also predicted island-wide population persistence under predicted sea level rise scenarios, but with O‘ahu’s largest gallinule populations losing >40% of current carrying capacity.

## Introduction

Island taxa are a conservation priority because of their high species endemism and elevated risks of extinction when compared to mainland ecosystems (Alcover, Sans & Palmer, 1998; Duncan & Blackburn, 2007; Kier et al., 2009). Avian extinctions on islands are among the best documented recent and pre-historic losses of vertebrate biodiversity (Olson & James, 1982; Duncan, Boyer & Blackburn, 2013), and extant island birds make up a large proportion of threatened avian taxa (Lee & Jetz, 2010). Climate change is a rapidly emerging threat to island species in general (Fordham & Brook, 2010) and birds in particular (Şekercioğlu, Primack & Wormworth, 2012). Among climate change threats to island species are lower adaptive capacity to environmental change (Buckley & Jetz, 2010), a reduced dispersal capacity to adjust to changing habitat conditions, a limited elevational or latitudinal gradient for such adjustments (of particular concern for birds, Devictor et al., 2008) and habitat inundation with sea level rise, a qualitatively higher risk for island systems (Mimura et al., 2007). Şekercioğlu, Primack & Wormworth (2012) emphasized that research on climate change impacts on tropical birds in particular is important because of a diverse array of likely impacts, and generally poor knowledge of the subject.

The Hawaiian archipelago is a hotspot for extinction, having lost the majority of its endemic avifauna to human impacts (Olson & James, 1982). Research has demonstrated climate change is having strong negative impacts on Hawaiian forest birds and seabirds (Atkinson & LaPointe, 2009; Reynolds et al., 2015), but very little attention has been paid to climate impacts on other endemic Hawaiian waterbird taxa. The Hawaiian common gallinule (‘Alae ‘ula, *Gallinula galeata sandvicensis; hereafter Hawaiian gallinule*), is among the most threatened of these (USFWS, 2011), with biannual population survey counts below 1,000 individuals (Reed et al., 2011) and a range limited to the islands of O‘ahu and Kaua‘i. We integrated data on vital rates, movement ecology, and climate change projections to generate a stochastic simulation model of the Hawaiian gallinule population on O‘ahu to investigate the potential impacts of management strategies and climate change on their extirpation risk. Hawaiian gallinules exhibited rapid population declines throughout the late 19th and early 20th centuries (Shallenberger, 1977; Banko, 1987; Griffin, Shallenberger & Fefer, 1990) due to hunting, habitat loss from wetland reclamation, and predation by exotic invasive species, and by the 1960s an estimated 60 individuals remained (Engilis & Pratt, 1993). Population increases have been achieved since the 1970s, principally attributed to the establishment of protected wetland refuges, predator control, and habitat management, by state and federal authorities (Reed et al., 2011; Underwood et al., 2013).

Hawaiian gallinules are one of many native Hawaiian bird species, including all of the waterbirds, that are conservation reliant (Reed et al., 2011; Underwood et al., 2013), requiring continuous management for populations to persist. Management for Hawai‘i’s endangered waterbirds typically includes control of mammalian, avian, and amphibian predators, regulating fresh water input to control depth and salinity, and regular removal of emergent vegetation through tilling, mowing, burning, or flooding to prevent habitat degradation and domination by exotic invasive plants (USFWS, 2011; VanderWerf, 2012). The distribution of gallinules on each island is naturally fragmented by the subspecies’ ecological specialization on limited coastal freshwater wetlands, with greater isolation caused by wetland loss from widespread anthropogenic landscape change (van Rees & Reed, 2014; van Rees et al., 2018b). Habitat patches (and therefore local subpopulations) on O‘ahu are generally small, most supporting fewer than 50 individuals at a site. This highlights the potential importance of dispersal for the persistence of an island’s population. Unfortunately, very little is known about Hawaiian gallinule movements, although a population genetic analysis by van Rees et al. (2018b) showed strong signs of genetic structure among gallinule populations on O‘ahu, indicating that movement may be restricted. This increasing awareness of the fragmented nature of O‘ahu’s gallinule population has led to suggestions for studying and improving connectivity between the island’s isolated subpopulations (van Rees & Reed, 2015; van Rees et al., 2018b). The cryptic behavior of Hawaiian gallinules has made field studies of their vital rates and movement behavior difficult; until recently, insufficient data were available to model population persistence for Hawaiian gallinules, or to evaluate alternative management scenarios and threat impacts.

Recent studies of the Hawaiian Islands have projected that climate change, particularly with respect to sea level rise, may have dramatic effects on Hawaiian coastal freshwater wetlands (Underwood et al., 2013; Kane et al., 2015a; Htun et al., 2016), raising additional concerns over the long-term viability of O‘ahu’s Hawaiian gallinule population. The freshwater wetlands upon which Hawaiian gallinules depend are found only along a narrow strip of flat, low-elevation land bordering the coastlines of the islands, and are therefore vulnerable not only to inundation with sea level rise but also to salinization (VanderWerf, 2012); as sea water rises, it can penetrate the freshwater aquifers that support many palustrine wetlands (Lau & Mink, 2006). Hawaiian gallinules appear to have the lowest tolerance for elevated salinity among Hawaii’s endangered waterbirds, so they may be threatened by habitat degradation from saltwater intrusion in addition to habitat loss from inundation with sea level rise (USFWS, 2011; Underwood et al., 2013).

These numerous sources of uncertainty and risk warrant quantitative assessment, and the US Fish and Wildlife Service’s (2011) Recovery Plan for Hawaiian Waterbirds lists population viability analysis (PVA) as a key part of the process toward de-listing this subspecies. PVAs are population models used to project population size and persistence into the future as quantitative assessments of extinction risk (e.g., Seal & Foose, 1989; Catlin et al., 2016). Depending on their structure, PVAs can incorporate a wide variety of demographic and life history information and various types of stochasticity to estimate probabilities of extinction or pseudo-extinction (the probability of declining below a threshold population size; Beissinger & Westphal, 1998; Morris & Doak, 2002). The objective of PVAs is making the most accurate projections or comparisons (among alternative management scenarios) possible using the best available data, which in the case of many declining or rare taxa are often very limited (Boyce, 1992; Morris & Doak, 2002; Zeigler & Walters, 2014). Beissinger & Westphal (1998) present guidelines for the responsible and practical use of PVA, stressing that their primary utility is in assessing relative impacts (rather than absolute predictions) and trade-offs among organism vital rates, associated management strategies, and their influences on extinction risk.

Recent studies have shown that a species’ behaviors can have major impacts on model predictions, in some cases showing higher (Gerber, 2006) and others lower (Grimm et al., 2005; Mortensen & Reed, 2016) extinction risk relative to a behaviorally uninformed model. Movement behavior is an especially important driver of population dynamics for small and fragmented populations, (Hanski, 1999; Maciel & Lutscher, 2013), mediating population connectivity (Taylor et al., 1993; Reed & Levine, 2005), which in turn may ameliorate extinction risk for small populations via the rescue effect (Brown & Kodric-Brown, 1977; Gotelli, 1991), recolonizing empty sites (e.g., Hanski, 1999), and genetic rescue (Keller & Waller, 2002).

The discrete distribution of Hawaiian gallinules on O‘ahu makes them an excellent study system for spatially explicit PVA (Walters, Crowder & Priddy, 2002), and their current existence in many small subpopulations warrants attention to the impacts of stochastic elements of population dynamics as well as to deterministic drivers of decline. The uncertainty in demographic parameter estimates for this taxon (van Rees et al., 2018a), and difficulty of studying them in the field means that vital rate sensitivity analysis could aid in prioritizing field study and data collection (Morris & Doak, 2002). The management dependence of this subspecies also raises questions about the efficacy of management alternatives. Finally, the threat of sea-level rise to Hawaiian gallinule populations, though referenced by several authors, has not been evaluated quantitatively. Here we create a spatially explicit population viability analysis for the Hawaiian gallinule on O‘ahu and examine extirpation risk in this subspecies both under current conditions and under potential climate-change scenarios and management alternatives.

## Materials & Methods

### Study area

**Figure 1:**
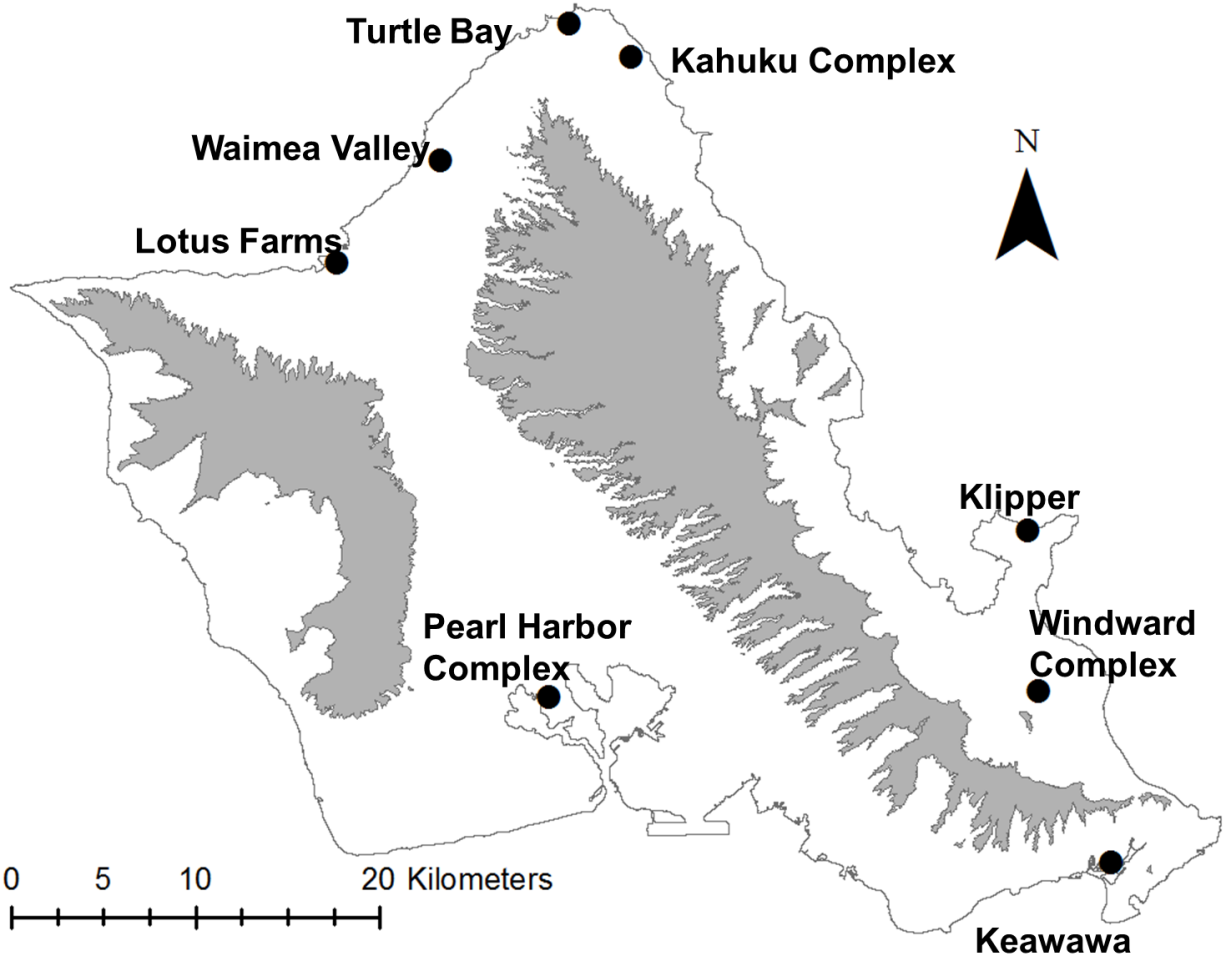
Map of study site (O‘ahu, Hawai‘i), highlighting locations of modeled populations of Hawaiian gallinules. Black dots indicate the approximate centroid of wetland habitats that make up a population; some complexes represent four or more separate wetlands, which were pooled based on population genetic information from Van Rees et al. (2018b). Gray areas represent the Waianae (left) and Ko‘olau (right) mountain ranges, which are presented for geographic and topographic reference.

We studied the population of gallinules on O‘ahu, Hawai‘i (21°28′N, 157°59′W), which persists in fragmented subpopulations along the island’s coastal plain (Fig. 1). Wetland habitats supporting breeding subpopulations of gallinules include state and federal wildlife refuges that are actively managed for waterbirds, gardens, agricultural areas, and golf courses. O‘ahu is the most developed island of Hawai‘i with the highest human populations, and it has experienced rapid landscape change (Giambelluca, 1986; Giambelluca, 1996; Klasner & Mikami, 2003), resulting in a highly diverse and increasingly urbanized landscape matrix. Most wetlands support small numbers of gallinules (five–30 individuals), although two larger wetland complexes may support subpopulations of ∼100 (C van Rees, 2018, unpublished data). To our knowledge, the O‘ahu population of gallinules is an effectively closed system, with no published records of movements between O‘ahu and Kaua‘i (Banko, 1987; van Rees et al., 2018a), although biannual waterbird surveys show extremely rare occurrences of gallinules on the Big Island, Maui, and Molokai, so the possibility of movement cannot be excluded. Demographic data used for this study were taken from van Rees et al. (2018a), and were collected from fourteen wetland sites on the island from different time periods from 1979–2017.

### Baseline population model

We used Vortex 10 (Lacy, 1993; Lacy & Pollak, 2017) to generate an individual-based stochastic simulation model of O‘ahu’s gallinule population. We chose this approach to explicitly model how the small size of many of our subpopulations affects extirpation risk, and the movement of individuals between subpopulations (Lacy, 2000; Walters, Crowder & Priddy, 2002). We used a time frame of 160 years, the span of ∼40 generations recommended by O’Grady et al. (2008) and Reed & Mccoy (2014), based on our estimated generation time of approximately four years as calculated in Vortex (using the Euler equation, Lacy, Miller & Traylor-Holzer, 2017). We set the duration of each year in days as 365 days. We defined a subpopulation as extirpated (or, in the software, the “extinction definition”) when only one sex remained; the same criterion was used for island-wide extirpation. We did not model population harvest or supplementation. Accordingly, the order of events in each Vortex year (as described in the program) was EV—Breed—Mortality—Age—Disperse—rCalc—Ktruncation—UpdateVars—Census. For each scenario, we ran 1000 iterations, and recorded the following metrics of extirpation risk for each subpopulation and for the total island population: (1) probability of extirpation by 160 years, (2) mean population size of extant populations (and subpopulations) at 160 years, and (3) deterministic and stochastic growth rates for that scenario. We did not include state variables as defined in the Vortex simulation model.

### Subpopulations and carrying capacity

We delineated subpopulations based on genetic evidence from van Rees et al. (2018b), combining subpopulations with no evidence of genetic structure (e.g., nonsignificant or near-zero *F*_ST_) into wetland complexes (Fig. 1). The carrying capacity of each subpopulation was estimated as the maximum recorded count of gallinules in the last 12 years, using data from both Hawai‘i’s biannual waterbird survey and playback surveys (following DesRochers, Gee & Reed, 2008) that we conducted for a separate project (C van Rees, 2018, unpublished data). The carrying capacities of wetland complexes were the sum of carrying capacities of their constituent wetlands (Table 1). The starting size (time = 0) of each subpopulation was set to 80% of K, which is similar to current estimated population sizes, and we assigned the model to start populations at a stable age distribution. We chose the default value (0.5) for correlation of environmental variance between subpopulations given an absence of quantitative data on this parameter and a general understanding that while habitats are geographically proximate due to the small size of the island, that precipitation conditions can differ dramatically based on orographic rainfall (Lau & Mink, 2006). We set the correlation between reproduction and survival to 0.1 due to repeated observations of failed nests and depredated broods with no evidence of increased adult mortality (C van Rees, pers. obs., 2018, M Silbernagle, USFWS ret., pers. comm., 2017).

**Table 1:** Sizes and size classifications of simulated Hawaiian gallinule subpopulations on O’ahu. Names, carrying capacities (*K*), and size classes of the eight subpopulations modeled in our study of the population viability of Hawaiian gallinules (*Gallinula galeata sandvicensis*), on O’ahu, Hawai’i.

Subpopulation name	*K*	Size class
Windward Complex	186	Large
Kahuku Complex	105	Large
Pearl Harbor Complex	40	Medium
Turtle Bay	38	Medium
Waimea Valley	27	Medium
Klipper	15	Small
Lotus Farm	14	Small
Keawawa	6	Small

For almost all wetland sites, gallinule counts from unpublished playback survey data yielded the highest numbers, and these values were consequently used. These counts likely have much lower detection error than waterbird survey counts (DesRochers, Gee & Reed, 2008), but our carrying capacities and starting population sizes nonetheless assume high detection and could be underestimates of true population size.

### Survival and reproductive system

We generated baseline model parameter values using information on reproduction and survival from van Rees et al. (2018a), with support and supplementation from data on related taxa (e.g., Common moorhen, *Gallinula chloropus*; Table 2). Even though Hawaiian gallinules do not breed until age 2, there is no evidence of age structure in survival rate beyond year 1 (van Rees et al., 2018a). Accordingly, we modeled survival using two age classes: hatch year (hatching to age 1), and after hatch year (age 1+ or adult). For adult (after hatch year, AHY) birds, we used the less conservative of two survival estimates generated by van Rees et al. (2018a), because it better accounted for the extremely poor detection in this taxon. We calculated first-year survival by combining survival rates to fledging with adult survival rates for the remainder of year 1 (10 months). We used information on fledgling survival from van Rees et al. (2018a), which reported ∼41% mortality within the 60-day period up to fledging; similar to observations in *G. g. cachinnans* in North America (Greij, 1994). Assuming adult mortality rates for the remaining 10 months of year 1, the total mortality in year 1 was estimated as 67% (Table 2). We estimated mean adult mortality to be 26.8%, using pooled data from van Rees et al. (2018a), and calculated variance in adult survival using the binomial equation (yielding a value of 3.3%), because the standard deviation from van Rees et al. (2018a) appeared to be inflated by detection errors. We chose a value of 15% for the variance in juvenile to reflect the large variability in chick survival observed in the field. The sensitivity of our model to this parameter estimate and those above was evaluated to assess the degree to which our assumptions and parameter uncertainty affect the outcome of the model and its predictions (see Sensitivity Analyses).

**Table 2:** List of vital rates, their values for our baseline population viability analysis, and range of values used for three sensitivity analysis methods. Letters in superscript indicate the reasons for which a given vital rate was chosen for sensitivity analysis; other parameters were not varied because they did not fit the criteria used. EV stands for environmental variation, the component of variance in vital rate value due to annual variation in environmental conditions. Vital rates that were selected for sensitivity analysis because of uncertainty are marked with^U^ and those selected because they are relevant to management are marked with^M^.

Parameter	Baseline value (SD)	Source	Perturbation	Conventional	Logistic regression
*Reproduction*					
Breeding system	Long-term Monogamy	Bannor & Kiviat (2002)	N/A	N/A	N/A
Min–Max age of reproduction (years)	2–10	Clapp, Klimkiewicz & Kennard (1982) and Van Rees et al. (2018a)	N/A	N/A	N/A
Distribution of broods per year^U,M^	0 to 4 by binomial dist’n	Nagata (1983), Gibbons (1986), Greij (1994); C van Rees (pers. obs., 2018)	N/A	±10% shift	N/A
Mean of brood size^M^	4.19 (1.82)	Van Rees et al. (2018a)	1–8, by 0.5	± 10%	1–8
Sex ratio at birth	1:1	Assumed	–	–	–
Percent females breeding^U^ ± EV	90 ± 10	C van Rees (pers. obs., 2018)	0–100, by 10	± 10%	–
Males in breeding pool	100%	Assumed	–	–	–
*Annual Mortality*					
Juvenile (HY) mortality ± EV^U,M^	0.67 (0.15)	Van Rees et al. (2018a)	0–1, by 0.1	± 10% (EV: ± 10%)	0–1 (EV: 10–25)
Adult mortality ± EV^M^	0.27 (0.033)	Van Rees et al. (2018a)	0–1, by 0.1	± 10% (EV: ± 10%)	0.02–0.66 (EV: 0.03–0.2)
*Population parameters*					
Carrying capacity ± EV^M^	See Table 1; ± 10%	Waterbird surveys; C van Rees (2018, unpublished data)	5–100 individuals, by 5	± 10% per population	4–25
Dispersal rates^U,M?^	See Supp. Materials	Van Rees et al. (2018b)	2–100× baseline, by 5	± 10%	2–80

We used the long-term monogamy mating system in Vortex, given observations of two closely monitored pairs (van Rees et al., 2018a) and general knowledge of the species (Bannor & Kiviat, 2002). We set the age of first breeding for males and females to 2 years (van Rees et al., 2018a). We used a maximum age of 10, which is the oldest observed Common gallinule (*Gallinula galeata*) from mark-resighting data in the United States (Clapp, Klimkiewicz & Kennard, 1982); Hawaiian gallinules have not been recorded as living this long (van Rees et al., 2018a). We assumed no reproductive senescence, since no evidence of this has been observed in Hawaiian gallinules (van Rees et al., 2018a). Because we had no information on the potential impacts of inbreeding depression in Hawaiian gallinules, and because it is considered of potentially lower importance for island bird species (Duncan & Blackburn, 2007) we did not include it in our model.

We defined offspring in the model as chicks, and used 8 as the maximum number of progeny per brood, which is the highest observed brood size on O‘ahu (van Rees et al., 2018a). We specified the distribution of brood sizes based on data from 103 broods on O‘ahu collated by van Rees et al. (2018a) (Table 2) and assumed a 1:1 sex ratio at hatch in the absence of evidence to the contrary. We set the maximum number of broods in a given year to 4, basing our estimate on field observations of two closely monitored pairs (van Rees et al., 2018a), as well as accounts of this and conspecifics under natural conditions (Benthum, 1931; Nagata, 1983). We modeled the distribution in number of broods produced per female per year using a binomial distribution with the estimate of nest success for O‘ahu (0.65; van Rees et al., 2018a) as the probability parameter, and the maximum number of broods in a year (4) as the number of trials (Table 2). Because Vortex does not accommodate additional modeling steps for nesting (and thus counts only chicks as offspring) our approach to modeling reproduction accounted for nest failure and hatch rates implicitly. Observed nest success rates were used to generate the number of successful broods per year, and our observed data on brood sizes rather than clutch sizes internalized hatch rates into reproduction in the model. We used sensitivity analyses of the distribution of brood sizes and mean brood size to examine the potential impacts of changing nest success rates as well as hatch rates on the outcomes of our model. Notably, the effects of these parameters could not be distinguished from uncertainty in clutch size, though this is one of our best-studied parameters (van Rees et al., 2018a), and we see little biological reason it should be changing across time or differing between subpopulations.

We did not make young dependent on their parents for multiple years, because Hawaiian gallinules can feed independently within a month of hatching (Chang, 2010). In the absence of evidence of polygyny, and assuming that all males of breeding age have the potential to compete for breeding vacancies, we set the percentage of breeding-age males included in the model’s breeding pool to 100%. All reproduction and survival parameters were identical between subpopulations, because we currently lack site-specific data on Hawaiian gallinule vital rates (van Rees et al., 2018a).

### Density dependence

Given observations that Hawaiian gallinules are aggressively territorial (Chang, 2010; C van Rees, pers. obs., 2018), and population models in other rails that took territoriality into account (Wanless, 2002; Hockey, Wanless & Brandis, 2011), we included density dependence in our model. We did this using the density dependence function provided by Vortex, which varies the probability of an individual female breeding in a given year based on the population size with respect to carrying capacity (Lacy & Pollak, 2017):

(1) \begin{eqnarray*}P \left( N \right) = \left( P \left( 0 \right) - \left[ P \left( 0 \right) -P \left( K \right) { \left( \frac{N}{K} \right) }^{B} \right] \right) \frac{N}{N+A} .\end{eqnarray*}

Here *P*(*N*) is the proportion of females that breed when the population size is *N*, *P*(*K*) is the proportion that breed when the population size has reached carrying capacity, and *P*(0) is the proportion of females breeding at low densities. We used a value of 0 for *A*, the Allee parameter, because we have seen no evidence for behaviors that would lead to Allee effects in this species (e.g., no dependence on group defense from predators; large habitats where finding mates would be difficult), and a value of 20 for the steepness parameter B, which made the function show few density dependent impacts until the population is above 0.8*K* (80% of carrying capacity). We assumed this high ceiling because the only published research on Hawaiian gallinules indicates that they are not food limited (DesRochers, SR & Reed, 2010). This study focused only on plant-based nutrition, which is the majority of the adult diet. We acknowledge, however, that the degree to which animal prey might possibly be limiting, especially at certain life stages (e.g., nesting or chick-rearing), has never been investigated. Empirical evidence for food limitation and food-driven density dependence in waterbirds appears to be restricted to dabbling ducks in boreal wetlands (e.g., Sjöberg et al., 2000; Elmberg et al., 2005), which are likely ecologically very different than our system, a highly cursorial rallid in tropical wetlands. Accordingly, given the best available knowledge of our system, we assumed that Hawaiian gallinules would likely not experience density dependent negative feedback until territorial behaviors (e.g., chasing and fighting) began negatively affecting survival and reproductive success. We set *P*(0), the baseline probability of a female breeding in a year at 0.90, and *P*(*K*), the probability of a female breeding under maximum density dependence, at 0.33. We estimated *P*(*K*) based on our observations that large family groups in densely populated wetlands typically had at most four non-breeding adult helpers, of which 2 were likely females assuming a 1:1 sex ratio, which implies that 1 in 3 adult females per territory would be breeding at high density (C van Rees, pers. obs., 2018).

### Dispersal

We estimated dispersal using unidirectional gene flow data from van Rees et al. (2018b) calculated using software MIGRATE (Beerli & Felsenstein, 1999; Beerli & Felsenstein, 2001). We estimated the number of migrant individuals per generation (*m*) from one wetland to another as *m* − *Mμ*, where *M* is the effective emigration rate between one subpopulation and another, and µis the mutation rate. We used *μ* = 10^−4^, which is a standard mutation rate for microsatellite dinucleotide repeats (Vigouroux et al., 2002; Marriage et al., 2009). We then converted the resulting per-generation estimate to per-year using Hawaiian gallinule generation time (four years) and to a percentage, to determine the percent of individuals from each wetland moving each year to each of the other wetlands. We used gene flow estimates to estimate movement rates instead of measures of genetic differentiation (e.g., *F*_ST_) because of their ability to provide separate values for different directions between the same subpopulation pair, and because fewer assumptions were made in converting those estimates to actual probabilities of movement (Beerli & Felsenstein, 1999; Beerli & Felsenstein, 2001; Whitlock & McCauley, 1999). We input these values as a matrix of pairwise inter-wetland percentage movement rates, included both males and females as dispersing sexes in the absence of evidence for sex-biased dispersal, and set the age range for dispersal as one to five years, based on observations from mark-resight studies (van Rees et al., 2018a). We estimated high percent survival of dispersers (95%) given our current understanding that gallinules likely disperse at night to avoid predation and fly at higher altitudes where they are unlikely to be struck by vehicles (van Rees et al., in press).

### Catastrophes

Generally, wetlands on O‘ahu are subject to few catastrophic events (typically, hurricanes), which we view as unlikely to have significant effects on survival or reproduction of Hawaiian gallinules. Observations indicate that adult Hawaiian gallinules are unlikely to be killed by flood events on O‘ahu (M Silbernagle, USFWS ret., pers. comm., 2017), although flooding is more common and possibly a greater threat on Kaua‘i (Htun et al., 2016; K Uyehara, USFWS, pers. comm., 2018) Hawaiian gallinules can re-nest quickly (van Rees et al., 2018a), indicating that nest and chick losses due to storm-related flooding might be quickly compensated by repeated breeding attempts. Additionally, Hawaiian gallinules are aseasonal breeders, apparently breeding year-round (DesRochers et al., 2009), so even widespread losses at a single time point would represent reproductive loss for only a small portion of the total annual breeding window, rather than disrupting a limited breeding season. Three major hurricanes have directly hit the main Hawaiian Islands with enough proximity to affect O‘ahu in the last 68 years (Central Pacific Hurricane Center, 2017). We generated a per-year probability using this value (0.04). Because subpopulations are spread across three different coasts of the island, we estimated that a hurricane could cause total reproductive failure due to flooding to a maximum of 50% of the population. Due to their aseasonal breeding habits, however, such a catastrophe would probably eliminate only about one fourth (25%) of the year’s breeding attempts in affected subpopulations, given that the combined nesting, incubation, and fledging time of Hawaiian gallinules is around 90 days. This would result in reducing the reproductive output of 50% of the island’s subpopulations by 25%, so we estimated that a hurricane would reduce reproduction by 12.5% in the year that it struck. We set catastrophes to reduce survival by 5% to account for the possibility of a small number of individuals being killed by flooding or during dispersal from flooded areas.

### Sensitivity analyses

We followed the sensitivity analysis protocols of Mortensen & Reed (2016), who conducted sensitivity testing using three approaches: (1) perturbation analysis, (2) relative sensitivity or elasticity, and (3) the logistic regression approach (Cross & Beissinger, 2001). Their perturbation approach involves systematically changing a single parameter to see how much a parameter value can be changed until a population either declines to extinction or persists throughout the study period (if it is going extinct under baseline conditions). This method explores potentially extreme parameter values in order to ensure proper model behavior (e.g., to ensure that a modeled population can go extinct, or persist, given parameter values of sufficient magnitude) and to identify the threshold value (or set of values) at which model behavior changes from extinction to persistence (or vice versa). This threshold can be used to determine how close current estimated parameter values are to a major change in population behavior, and whether management or future conditions could possibly result in a shift between extinction and persistence We used perturbation analyses of subpopulation carrying capacity and dispersal rates as a test of two competing management strategies, wetland management and connectivity conservation, to assess their relative value as strategies for decreasing island population and subpopulation extirpation risk. In particular, we were interested in whether extirpation risk could be altered dramatically with realistic shifts in these parameters, or if the changes required are beyond the capability of current management. The logistic regression approach uses logistic regression to examine the relationship between the value of a given parameter and the probability of extinction given a large number of samples of possible parameter values and a binary outcome of extinct or not extinct at the end of the study period (McCarthy, Burgman & Ferson, 1995; Cross & Beissinger, 2001). Notably, each of these methods of sensitivity analysis offers a different advantage and yields different information about the model (Mortensen & Reed, 2016; Manlik, Lacy & Sherwin, 2017). The principle advantage of elasticity analysis is its comparability to other studies as a widely-used metric, while perturbation analysis reveals the points at which model behavior changes across parameter values, and provides perspective on whether system shifts (e.g., extinction vs. persistence) are feasible with realistic parameter values. The logistic regression approach allows for the examination of each parameter’s effect on model outcome while taking the effects of all others into account (Cross & Beissinger, 2001; Mortensen & Reed, 2016). We performed sensitivity analyses on all subpopulations separately, and on the entire O‘ahu population combined.

We performed perturbation analyses, following Reed, Murphy & Brussard (1998), on mean and variance of juvenile and adult mortality, percentage of females breeding in a given year, carrying capacity, mean brood size, and dispersal rate (Table 2). We varied both survival parameters from 0-1 in increments of 0.1, and percentage of females breeding from 0-100% in increments of 10%. In order to simulate habitat management, we varied K from 5-100 in increments of 5 for all subpopulations that had a probability of subpopulation extirpation >0 in our baseline model, which included all populations other than the Windward and Kahuku complexes (Fig. 1). We did not increase the carrying capacities of subpopulations with ∼0% extirpation risk under baseline conditions, because no changes in carrying capacity could further reduce extirpation risk in these subpopulations. We varied the distribution of brood sizes (which we used as a proxy for reproductive success) based on a normal distribution with a mean that we varied between 1 and 8 chicks (standard deviation of 1), encompassing the range of observed values on O‘ahu (van Rees et al., 2018a), and changed mean brood size by 0.5 chicks at a time. We changed dispersal rate by applying a multiplier across all inter-wetland movement rates, thus keeping relative dispersal rates the same and increasing overall movement and population connectivity on O‘ahu. We varied this multiplier from 2 to 100, effectively varying annual probability of individual dispersal by two orders of magnitude, but maintaining relative rates. We used this broad range to reflect current uncertainty about the extent to which connectivity might be altered by management, and uncertainty over the true mutation rate of microsatellite markers used to estimate per-generation movement rates. Each scenario was run for 1000 iterations and 160 years.

We conducted elasticity analysis by changing each parameter by ±10% of its mean value, and dividing the difference between the stochastic lambda (*λ*) of positive and negative scenarios by 0.2 times the stochastic lambda of the baseline scenario, according to the equation (*λ*_+_ − *λ*_−_)∕(0.2∗*λ*_0_) (Cooper, Walters & Priddy, 2002), where *λ*_+_ and *λ*_−_ are the positive and negative scenarios, respectively, and *λ*_0_ is the baseline scenario. We calculated stochastic lambda using the stochastic instantaneous growth rate (*r*) provided by Vortex for each scenario (using *λ* = *e*^*r*^). We assessed the relative sensitivity of our modeled populations to mean and variation of juvenile and adult mortality, the distribution of number of broods per female per year, the average brood size, the percentage of breeding-aged females breeding in a year, the carrying capacity K of each subpopulation, and population connectivity (Table 2). We varied the distribution of the number of broods per year by subtracting 10% of the proportion of brood numbers falling in each category (zero, one, two, three, and four broods per year) and adding it either to the next highest or lowest category, depending on the direction being tested. Accordingly, the distribution was shifted to higher or lower values by 10%. We called this parameter “distribution of no. broods per year”, which represents the probability distribution of the number of broods per year a simulated pair might have (i.e., the probability of having zero, one, two, three, or four successful nests) given that they attempted to breed. In our sensitivity analysis for this parameter, we changed the distribution by adjusting the frequency of higher vs. lower numbers of broods per year. We changed the distribution of brood sizes per year by modeling brood size as a normal distribution, and adding or subtracting 10% from the mean value. We altered dispersal rate by adjusting the overall dispersal multiplier by ±10%. We used Cooper, Walters & Priddy (2002)’s rule of thumb for assessing the relative sensitivity of model outcomes to changes in parameter values, whereby any parameter with a sensitivity value of >1 or <−1 was considered to have a disproportionate effect on population growth rate.

For logistic regression analysis, we used Latin Hypercube sampling in Vortex to randomly generate parameter sets selected from uniform distributions that we determined using observed and feasible values for Hawaiian gallinules. We ran 10 iterations for each parameter set, resulting in 10,000 total simulations for regression analysis. We then performed logistic regression using the ‘car’ package (Fox & Weisberg, 2011) in R 3.2.2 (R Core Team, 2015), treating extirpation (or persistence) at 160 years as the dependent variable. We conducted logistic regression using carrying capacity, mean juvenile and adult mortality, environmental variation in juvenile and adult mortality, percentage of females breeding, and connectivity as explanatory variables. Each parameter was varied according to a uniform distribution, bounded where appropriate based on knowledge of feasible values. We varied mean juvenile mortality rate from 0–1 because of our extremely poor knowledge of the parameter and its large apparent variation in the field, and adult mortality from 0.02–0.65, based on the potential range of annual adult apparent mortality estimates found in van Rees et al. (2018a). We varied the dispersal multiplier from 2 to 80, again reflecting poor knowledge of possible values (Table 2). We combined these parameters as predictor variables in a single generalized linear model with probability of extirpation (PE) as the response variable. We compared the explanatory value of different parameters using their standardized regression coefficients, calculated by dividing the regression coefficient by its standard error.

### Climate scenarios

We used readily available spatial data on sea level rise and maps of the location and extent of modeled Hawaiian gallinule habitats to estimate the reduction in habitat area expected from future sea level rise on O‘ahu. Wherever existing habitat features overlapped with future areas of inundation (indicated by the distribution of future inundated areas), we counted that area as habitat loss. We assumed that reductions in habitat would correspond with a proportional reduction in carrying capacity; in other words, that gallinule population densities are uniform throughout their habitats. The spatial data that we used to approximate sea level rise on O‘ahu were created by the National Oceanic and Atmospheric Administration’s (NOAA) Office of Coastal Management, and are available through their sea level rise data portal (https://coast.noaa.gov/slrdata/). These sea level rise projections do not account for the impacts of erosion, island subsidence, wetland migration through accretion, or human management for sea level rise mitigation, but represent a baseline model of potential habitat losses due to inundation. We used these models to generate an estimate of the potential magnitude of reduction of gallinule habitat and carrying capacity on O‘ahu under projected sea level rise scenarios, and to gauge the relative threat of sea level rise compared to other potential factors affecting extirpation risk in this taxon.

We estimated changes in habitat area (carrying capacity) at two scenarios, 0.914 m (3 feet, hereafter ∼1 m) and 1.829 m (six feet, hereafter ∼2 m), given a limited number of scenarios for which data were available on the NOAA sea level rise portal. These values correspond approximately to the range of sea level rise projected by Vermeer & Rahmstorf (2009) for the year 2100, which other researchers have found to be more predictively robust than the IPCC (2007) projections (Rahmstorf, Perrette & Vermeer, 2011; Kane et al., 2015a). Notably, Vermeer and Rahmstorf’s (2009) estimates are only for a 100-year projection, so we chose the highest available sea level rise value from available NOAA maps (1.829 m) for our 160 year time frame, representing a highly conservative estimate. Our smaller value (0.914 m) corresponds to a conservative value for an 80-year projection (Vermeer & Rahmstorf, 2009).

We combined our own maps of gallinule habitats on O‘ahu with data from the U.S. Fish and Wildlife Service’s National Wetlands Inventory (USFWS, 2017) to generate outlines of the habitats supporting all subpopulations modeled in this study. We converted these polygon maps to 3m resolution rasters using the Polygon to Raster tool in ArcMap 10.4.1 (ESRI, 2012), and for each subpopulation, added this map to one of the two sea level rise maps using the raster calculator. We then calculated the area of overlap to estimate the proportion of habitat pixels that would be inundated with salt water. Using this value as an ending carrying capacity at either 80 or 160 years, we designed a power function of the form aX^*b*^ to approximate the shape of the sea level rise curve depicted in IPCC (2007) and Vermeer & Rahmstorf (2009). We applied this to the carrying capacity of affected populations in Vortex to approximate the change in carrying capacity across time according to (2):

(2) \begin{eqnarray*}{K}_{t}={K}_{0}- \left[ {K}_{0}\ast L \left( \frac{a{t}^{b}}{Y} \right) \right] ,\end{eqnarray*}

where *K*_*t*_ is carrying capacity at time *t*, *K*_0_ is the original carrying capacity, *L* is the total proportion of habitat area lost at year 80 or 160 (depending on the simulation), *t* is the current year, *Y* is the last year of the simulation (80 or 160), and *a* and *b* are shape parameters used to approximate the pattern of projected global sea level rise. We determined values of *a* and *b* separately for 80 year and 160 year scenarios to maintain curve shape while passing through a different point at *t* = 80 or *t* = 160 (for 80 years, *a* = 0.06 and *b* = 1.613; for 160 years, *a* = 0.02 and *b* = 1.7385). We ran sea level rise scenarios using baseline model parameters. Carrying capacities of individual subpopulations were altered independently according to separate analyses of their potential area loss. Both scenarios were run with 1,000 iterations, with the ∼1 m scenario projecting for 80 years, and the ∼2 m scenario for 160 years (Table 3).

**Table 3:** Scenarios for population viability analysis tested in our population projection model of Hawaiian gallinules on O’ahu. Restoration/creation of habitat involved hypothetical management options increasing carrying capacity at small and medium wetlands, and connectivity involved increasing overall connectivity by increasing the multiplier of baseline dispersal rates between wetlands. The two sea level rise scenarios have reduced *K* over time according to an equation approximating common projections of sea level rise in 80 and 160 years, respectively. Parameter change represents the amount that each parameter was altered for a given scenario. In the case of sea level rise scenarios, this value represents the maximum reduction in *K* experienced during the scenario, achieved at the end of the scenario (represented by *L* in Eq. (1)).

Scenario	Parameter change	Time frame (years)
**Baseline**	N/A	160
**Restoration/creation**	As per Table 2	160
**Connectivity**	As per Table 2	160
**Sea level rise (∼1 m, 80 year)**		80
Windward Complex	0.94^∗^ *K*^a^	
Kahuku Complex	0.83^∗^*K*^a^	
Pearl Harbor Complex	0. 89^∗^ *K*^a^	
**Sea level rise (∼2 m, 160 year)**		160
Windward Complex	0.46^∗^ *K*^a^	
Kahuku Complex	0.49^∗^ *K*^a^	
Turtle Bay	0.01^∗^ *K*^a^	
Pearl Harbor Complex	0.00^∗^ *K*^a^	


**Notes.**

a Distributed according to Eq. (1) over the total time frame.

## Results

### Model projections

Our baseline model showed that O‘ahu’s island-wide Hawaiian gallinule population has a high likelihood of persistence over the 160 year time frame (Table 4). Probability of extirpation varied strongly between subpopulations, however, with the large subpopulations having probabilities near 0, and the small populations having probabilities near 1.0. Among these small subpopulations, median times to extirpation ranged from four to seven years and mean times from five to 8.9 years. The longest times to extinction among any iterations for small populations were on the order of 50 years. Medium subpopulations with nonzero probabilities of extirpation had mean times to extirpation ranging from 55.5–87.7 years; the median time to extirpation for Waimea valley was 53 years. The extirpation of small subpopulations appears to drive the very slight decreases in mean estimated island-wide population size at year 160, though average increases in population size in medium and large populations compensate for much of this loss. The stochastic population growth rate (*r*) for the total island population was 0.25, indicating rapid growth, although this ranged from 0.09 to 0.24 in different subpopulations, where larger subpopulations exhibited higher rates.

**Table 4:** Comparison of population viability of Hawaiian gallinule overall island population and subpopulations on O’ahu across management and sea-level rise scenarios. Probability of extirpation is the probability that a given (sub) population was extirpated over all simulations of a scenario, and mean *r* is the mean stochastic growth rate of a population for the scenario. The mean ending population size is the mean number of individuals left in a population when that population was extant at the end of a simulation, and the percent of starting population at ending time is the proportion of the starting population represented by the mean ending population size.

Scenario	Probability of extinction	Mean *r* (SD)	Mean ending population size (SD)	Percent of starting population at ending time
**Baseline**	**0.0**	**0.25 (0.38)**	**336 (46)**	**97%**
Windward Complex	0.0	0.24 (0.51)	171 (25)	115%
Kahuku Complex	0.0	0.23 (0.52)	96 (17)	115%
Pearl Harbor Complex	0.05	0.18 (0.51)	36 (11)	111%
Turtle Bay	0.08	0.18 (0.51)	33 (11)	109%
Waimea Valley	0.79	0.16 (0.51)	22 (10)	100%
Klipper	0.99	0.11 (0.55)	5 (5)	37%
Lotus Farm	0.99	0.09 (0.55)	6 (4)	43%
Keawawa	0.99	0.12 (0.58)	3 (1)	64%
**Sea level rise** (∼1 m, 80 year)	**0.0**	**0.26 (0.37)**	**311 (35)**	**89%**
Windward Complex	0.0	0.24 (0.50)	160 (23)	107%
Kahuku Complex	0.0	0.23 (0.51)	80 (13)	95%
Pearl Harbor Complex	0.06	0.19 (0.51)	31 (7)	96%
Turtle Bay	0.00	0.19 (0.51)	32 (7)	106%
Waimea Valley	0.69	0.16 (0.51)	20 (7)	91%
Klipper	0.99	0.09 (0.54)	9 (7)	75%
Lotus Farm	0.99	0.07 (0.54)	5 (3)	35%
Keawawa	0.99	0.10 (0.56)	3 (2)	75%
**Sea level rise (∼2 m, 160 year)**	**0.0**	**0.25 (0.38)**	**150 (18)**	**43%**
Windward complex	0.0	0.24 (0.50)	90 (14)	60%
Kahuku Complex	0.0	0.23 (0.51)	53 (9)	63%
Pearl Harbor Complex	0.06	0.16 (0.51)	5 (2)	16%
Turtle Bay	0.00	0.16 (0.51)	5 (2)	17%
Waimea Valley	0.69	0.16 (0.51)	22 (10)	100%
Klipper	0.99	0.10 (0.55)	9 (7)	75%
Lotus Farm	0.99	0.09 (0.55)	3 (1)	21%
Keawawa	0.99	0.10 (0.56)	2 (0)	50%

### Model sensitivity

**Figure 2:**
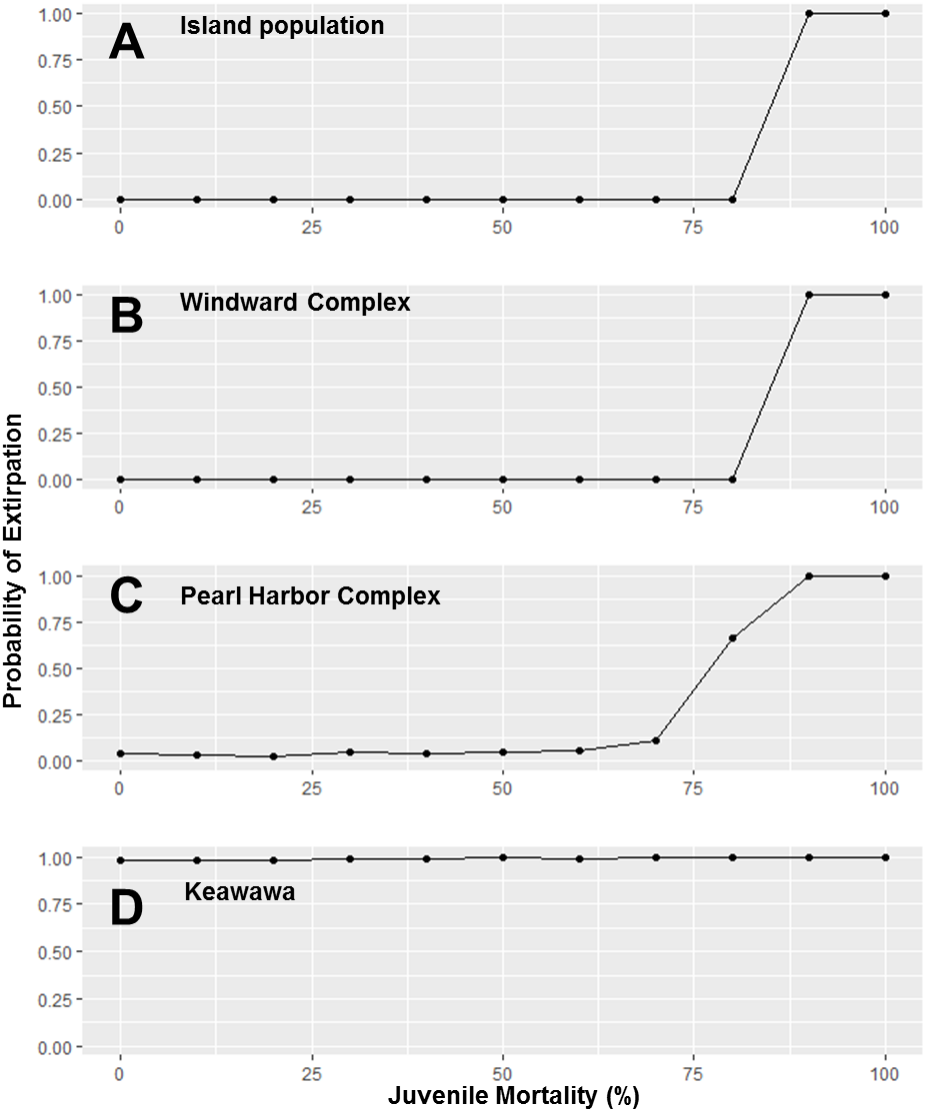
Perturbation analysis of juvenile mortality on probability of extirpation for the O‘ahu population and Windward, Pearl Harbor, and Keawawa subpopulations of Hawaiian gallinules. Perturbation analysis examining the sensitivity of extirpation probability among Hawaiian gallinules populations on O‘ahu to varying levels of juvenile mortality. Results are shown for the overall island population (A) and three wetlands whose sensitivity is representative of other wetlands of their size class (in descending size from B–D). Points represent tested parameter values.

**Figure 3:**
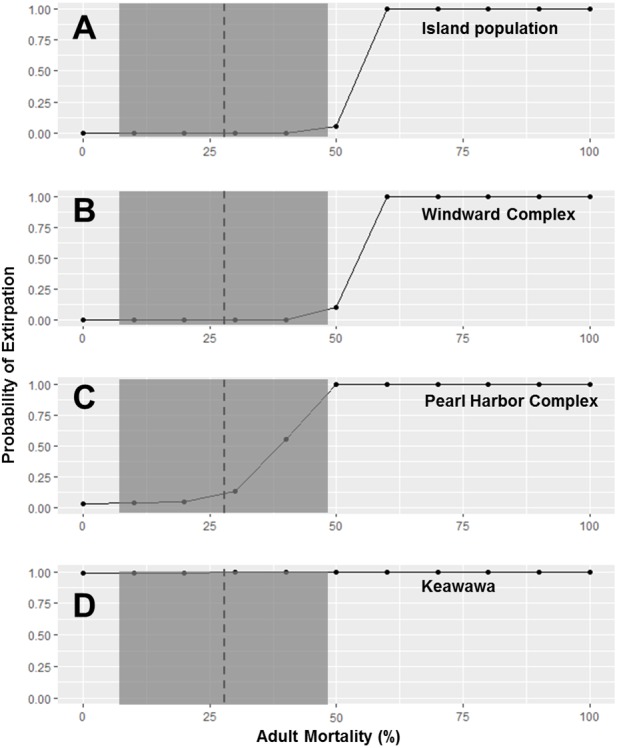
Perturbation analysis of adult mortality on probability of extirpation for the O‘ahu population and Windward, Pearl Harbor, and Keawawa subpopulations of Hawaiian gallinules. Perturbation analysis examining the sensitivity of extirpation probability among Hawaiian gallinule populations on O‘ahu to varying levels of adult mortality. Results are shown for the overall island population (A) and three wetlands whose sensitivity is representative of other wetlands of their size class (in descending size from B–D). The dashed vertical line and shaded boxes indicate the mean survival estimate ± SD from Van Rees et al. (2018a).

**Figure 4:**
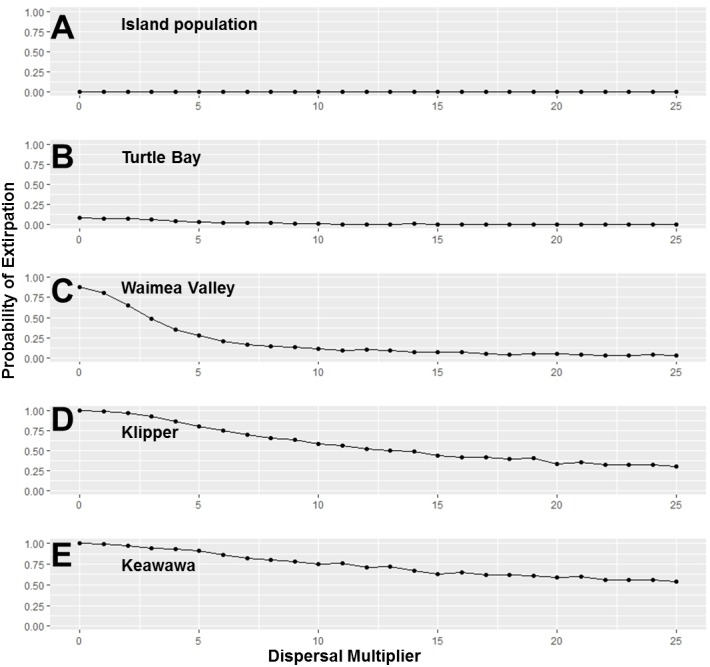
Perturbation analysis of dispersal rate (connectivity) on probability of extirpation for the O‘ahu population and the island’s four smallest subpopulations of Hawaiian gallinules. Perturbation analysis examining the sensitivity of extirpation probability among Hawaiian gallinules populations on O‘ahu to varying levels of inter-wetland dispersal rates. This perturbation analysis represents the range of impacts possible under management scenarios in which connectivity among subpopulations is increased. Results are shown for the overall island population (A) and the four smallest subpopulations, in descending order (B–E). Points represent tested parameter values.

**Figure 5:**
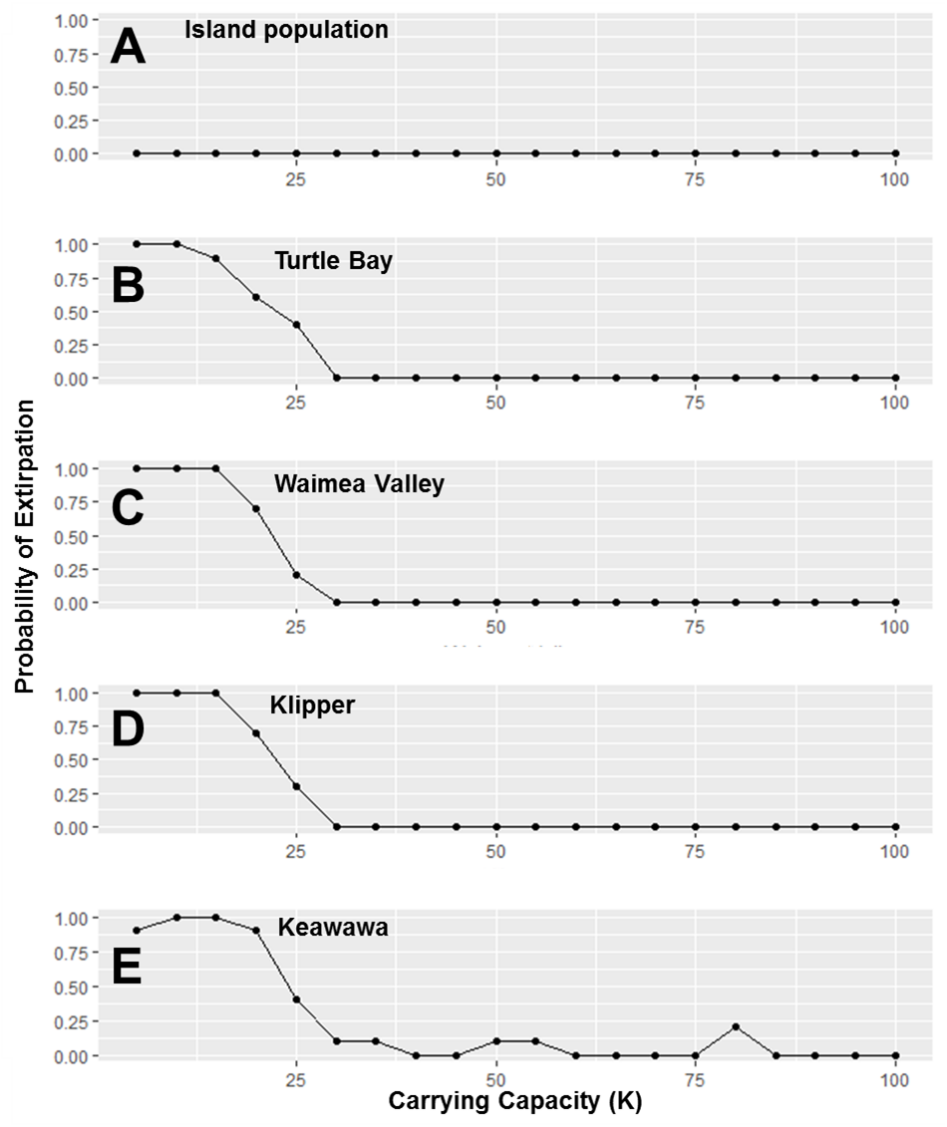
Perturbation analysis of carrying capacity on probability of extirpation for the O‘ahu population and the island’s three smallest subpopulations of Hawaiian gallinules. Carrying capacity (*K*) values simulate hypothetical changes to carrying capacity due to habitat management. Results are shown for the overall island population (A) and four wetlands whose sensitivity is representative of other wetlands of their size class (in descending size B–E). Points represent tested parameter values.

Because subpopulations of similar sizes showed similar trends in stochastic population growth rate and probability of extirpation, we do not show results for all subpopulations, but instead show representative examples of each size class (Figs. 2–5). Perturbation analysis showed that large and medium subpopulations transitioned rapidly from low probabilities of extirpation to high (*P* =  ∼ 1.0) probabilities when juvenile mortality rose above 80% (Fig. 2). Perturbation of adult mortality showed a transition point for large subpopulations from low to high probability of extirpation at 50% mortality, although this point shifted toward lower values in medium-sized subpopulations (Fig. 3). Small subpopulations’ extirpation probabilities remained at 1.0 for all values of juvenile and adult survival. The range of parameter uncertainty for adult survival encompassed values that were meaningful for medium-sized subpopulations (i.e., there were large differences in probability of extirpation across values ±1 SD from our parameter estimate), but this was not the case for large or small subpopulations.

Large subpopulations and the overall island population transitioned from near 0 extirpation probability to near 1.0 extirpation probability when the percent of females breeding decreased below 30% (Fig. S4). This transition was more gradual for medium subpopulations, which increased steadily from about 70% of females breeding, reaching an extirpation probability near 1.0 at 30%. For mean brood size, large subpopulations and the overall island population had extirpation probabilities near 1.0 when mean brood size was zero, but decreased to an extirpation probability of ∼0.0 when mean brood sizes were one or higher (Fig. S5). Medium-sized subpopulations had a probability of extirpation near 1.0 for mean brood sizes of zero and one, but declined to ∼0.25 with a mean brood size of two, and remained near zero at all higher values. Extirpation probability of small populations remained at 1.0 for all simulated values of percent of females breeding and brood size.

Our investigation of management scenarios in which connectivity was increased showed that with large (e.g., 10 to 25 times) increases in the dispersal multiplier, the extirpation risk of small subpopulations could be reduced by 40–75% (Fig. 4), but that it had little to no effect the extirpation risk of medium and large subpopulations, or the overall island population. The probability of extirpation of the two smallest subpopulations, Klipper and Keawawa, declined rapidly with increases of two to 20 times current dispersal, and showed slower declines after that point. For medium-sized subpopulations, extirpation risk declined sharply from two to 12 times detected dispersal rates, at which point probability of extirpation was near zero. For the habitat management scenario, perturbation of the carrying capacity of small and medium subpopulations showed consistently that carrying capacities above 15 individuals led to rapid decline in extirpation probability, reaching 0 by around ∼30 individuals (Fig. 5).

Conventional, relative sensitivity analysis showed that none of the tested vital rates had a disproportionate (>1.0) effect on stochastic annual population growth rate (*λ*). Juvenile mortality had the largest effect on the island-wide population persistence (−0.61; Table 5), though its effects were smaller for small subpopulations (e.g., −0.46 for Klipper). Mean brood size (a proxy for reproductive success, including nest failure and hatch rates) and percent females breeding had the next largest effects, with 0.30 and 0.23, respectively, with greater sensitivity to brood size among small subpopulations, and greater sensitivity to percentage of females breeding among large subpopulations. Adult mortality had a moderate effect on population growth rate (−0.17), which was greater for smaller subpopulations (e.g., −0.30 for Keawawa and −0.54 for Lotus Farm). Dispersal rate had a negligible effect on *λ* for the island-wide population, but small populations showed sensitivities up to 0.14. Environmental variation in adult and juvenile mortality and carrying capacity both also had little effect on *λ*, with slightly stronger effects (e.g., 0.03–0.06) on small subpopulations. Our logistic regression analysis showed that mean juvenile mortality, mean adult mortality, and mean brood size accounted for the most variability in observed extirpation probability of the overall island population (Table 5). Variance in juvenile mortality accounted for a greater proportion of variability than did variance in adult mortality, and both carrying capacity and dispersal rate explained very little variation. The importance of dispersal and carrying capacity was much larger for small subpopulations, with standardized coefficients as much as seven times larger than those for the island-wide population (e.g., Keawawa, −21.73) for dispersal, and three times higher for carrying capacity. The p values for all covariates in our logistic regression model were statistically significant (*p* < 0.0001 in all cases).

**Table 5:** Sensitivity of probability of extinction (PE) and stochastic population growth (*λ*) of the overall population of Hawaiian gallinules (*Gallinula galeata sandvicensis*) on O’ahu, Hawai’i to changes in various model parameters. “Dist’n of no. broods per year” represents the probability distribution of brood sizes (zero, one, two, three, and four) for individuals in the simulation. The starting probability distribution was based on a binomial distribution using observed values for nest success and the maximum number of broods per year (see Methods). Population growth was most sensitive to mean juvenile mortality and mean brood size, while PE was most sensitive to mean juvenile and adult mortality, mean brood size, and variance in adult mortality.

Parameter	Sensitivity to *λ*	Sensitivity to PE
Mean juvenile mortality	−0.61	40.47
Variance in juvenile mortality	0.01	10.54
Mean adult mortality	−0.17	38.15
Variance in adult mortality	0.00	12.69
Dist’n of no. broods per year	0.12	–
Mean brood size	0.30	−30.85
Percent females breeding	0.23	–
Carrying capacity	0.02	−5.28
Dispersal rate	0.00	−3.83

### Sea level rise scenarios

**Figure 6:**
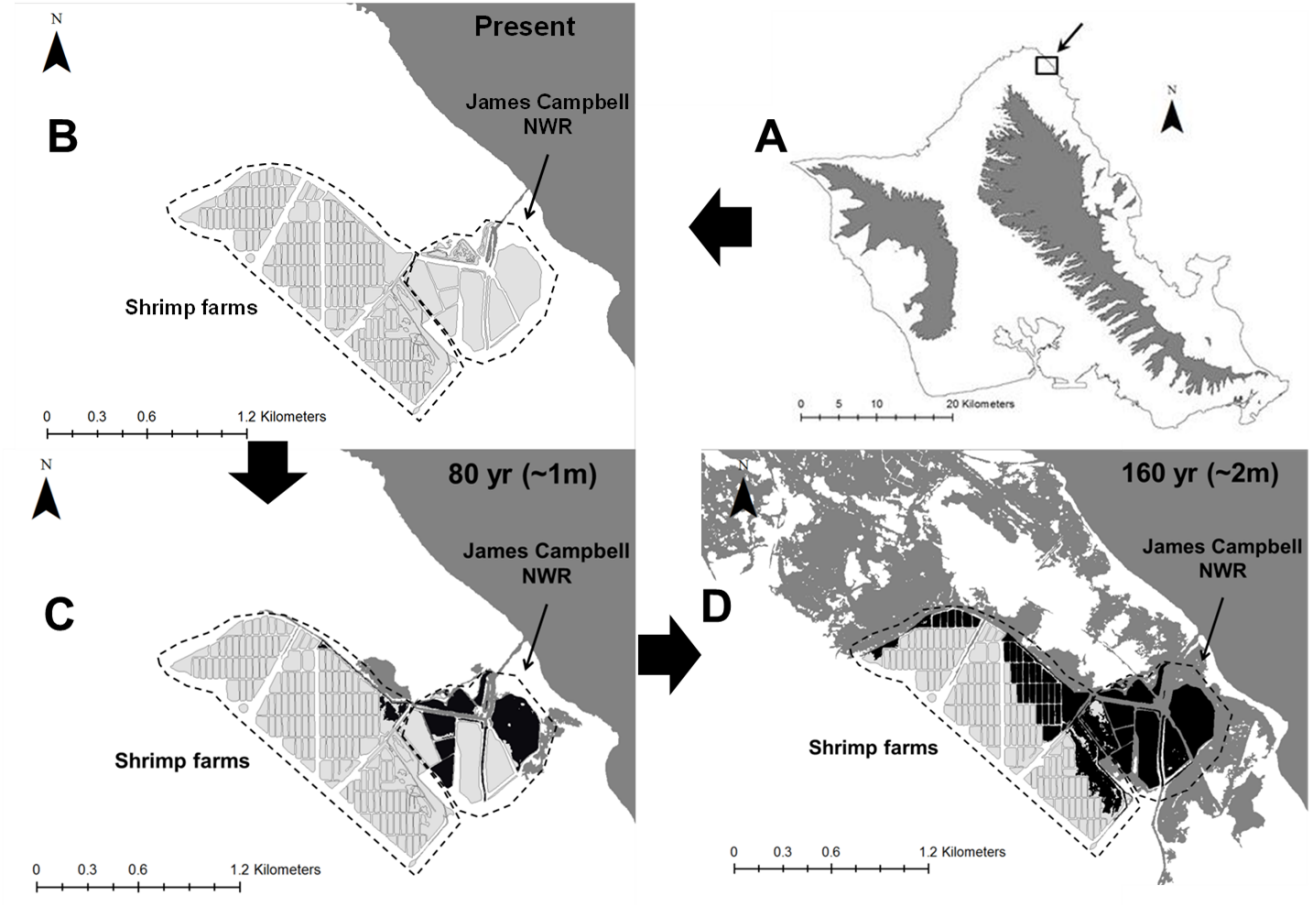
Spatial representation of potential Hawaiian gallinule habitat loss due to sea level rise over 80-year and 160-year time scales at the Kahuku wetland complex on the North Shore of O’ahu. The location of the wetland complex is shown in (A), and current habitat distribution is displayed in (B). Light gray areas indicate current Hawaiian gallinule habitat, dark gray indicates areas inundated with seawater (in (B), this represents current coastline). Black areas indicate current Hawaiian gallinule habitat lost due to seawater inundation. (C) ∼17% of habitat in the Kahuku complex is lost with ∼1 m sea level rise (modeled here as 80 years). (D) >50%, including the areas of highest gallinule density, may be inundated with ∼2 m sea level rise (modeled here as 160 years).

Our Hawaiian gallinule habitat maps encompassed ∼430 ha of wetland habitat on O‘ahu. In the 80 year (∼1 m sea level rise) scenario, a total of 36 ha of wetland habitat (8% of total) are predicted to be lost due to salt water inundation, with a sharp increase in the 160 year (∼2 m sea level rise) scenario, in which 239 ha (56%) are predicted to be lost. Due to their landscape context, four wetlands (Keawawa, Klipper, Lotus Farm, Waimea Valley) were unaffected by projected sea level rise in either scenario, and Turtle Bay was unaffected in the 80-year scenario. In contrast, the Windward, Kahuku, and Pearl Harbor Complexes are predicted to lose 5.6%, 17%, and 11%, respectively, of their total carrying capacity in the 80-year scenario. In the 160 year and ∼2 m sea level rise scenario, the Windward Complex is predicted to lose 54% of its carrying capacity, the Kahuku Complex 51% (Fig. 6), the Pearl Harbor Complex ∼100%, and Turtle Bay 99%.

Simulated island-wide gallinule populations on O‘ahu had 0.0 probability of extirpation under both our 80-year and 160-year sea level rise scenarios, though the mean population size at the end of the simulation was 11% lower than the starting size at 80 years, and 57% lower in 160 years (Table 4). Probabilities of subpopulation extirpations were not changed by sea level rise at 80 years, although the ending population sizes of the three largest subpopulations were smaller than in the baseline scenario. Pearl Harbor and Turtle Bay had population declines in excess of 80% over the 160-year (∼2 m rise) timeframe, while the Windward and Kahuku complexes showed declines of 40% and 37%, respectively. The probabilities of extirpation of the Windward and Kahuku complexes remained approximately 0.0 after 160 years, but they increased dramatically for both Pearl Harbor (from 0.05 to 0.77) and Turtle bay (from 0.08 to 0.80). Small populations were equally likely to be extirpated during either sea level rise scenario as in the baseline scenario. Stochastic population growth rates showed little change between climate change scenarios, both for the overall island population and individual subpopulations.

## Discussion

We developed a spatially explicit, individually based, multi-population projection model for the Hawaiian gallinule on O‘ahu and assessed the relative effects of potential management strategies (via sensitivity analyses) and sea level rise scenarios on extirpation risk. This study contributes to a growing and important body of research on the population-level impacts of environmental change on the viability of wildlife populations (Johst et al., 2011). Population projections should generally be cautiously interpreted and used as tools for assessing relative risks rather than making absolute predictions (Beissinger & Westphal, 1998). Consequently, this study provides baseline estimates of the relative sensitivity of O‘ahu’s Hawaiian gallinule population to different management strategies and threats, using the best information currently available.

Under our baseline scenario, O‘ahu’s Hawaiian gallinule population had a high probability of persistence (extirpation probability of ∼0.0) over 160 years, with a positive stochastic growth rate, and slight population decline due to the extirpation of small populations. The growth rate emerging from our model parameter values indicates that the Hawaiian gallinule’s reproductive capacity makes them capable of quickly responding to habitat management and other improvements of local carrying capacity. In contrast, the high extirpation probabilities of the smaller subpopulations are of concern for the long-term viability of the population under present conditions. van Rees et al. (2018b) found that two of the island’s smallest subpopulations are also home to some of the most genetically distinct birds on the island, with several private alleles. The loss of these small populations would thus come with considerable losses in overall genetic variability for the subspecies. Given our model findings that extirpation of these subpopulations is highly likely within less than 50 years in the absence of management or increased dispersal, the loss of these small subpopulations and their unique contribution to the subspecies’ remaining genetic diversity seems likely. Our projections emphasize that island-wide persistence is largely dependent on the fate of several large- and medium-sized subpopulations on the island, because all small subpopulations and some medium-sized subpopulations had high probabilities of extirpation. An additional risk factor for the island’s smallest subpopulations (Keawawa and Klipper) is uncompensated emigration (Fahrig & Merriam, 1985), because estimated gene flow rates out of these subpopulations were much higher than rates into them (van Rees et al., 2018b), making emigration much more likely than immigration in our simulation. Such emigration, combined with stochasticity is likely what makes these subpopulations act as sinks in our simulation (Gyllenberg & Hanski, 1992; Lacy, 2000). Although these factors may play a role in actual population dynamics on the island, the degree to which they are affected by site-specific vital rates and dispersal probabilities is unknown.

Very little is known about the movement rates of Hawaiian gallinules. We made our estimates of dispersal between subpopulations based on gene flow data, which required us to estimate the mutation rates of microsatellite markers used by van Rees et al. (2018b). Consequently, after first-year survival, movement between subpopulations is the most uncertain parameter in our model. Additionally, dispersal rates estimated using gene flow models only detect those movements that led to breeding, and thus underestimate to an unknown degree the actual movement rates amongst O‘ahu’s subpopulations, which might have other demographic implications. Empirically based simulation models of other avian habitat specialists have shown that, even where long distance dispersal is rare, mortality rates during dispersal can have significant impacts on population projections (e.g., Cooper, Walters & Priddy, 2002). The potential for sex-biased dispersal rates is also of particular interest given the small size of many of O‘ahu’s subpopulations and the subsequent importance of stochasticity for their persistence. For example, Schiegg, Walters & Priddy’s (2002) spatially explicit, individual-based population model of the Red-cockaded Woodpecker (*Picoides borealis*) showed strong dispersal effects on population growth rate driven by sex-specific differences in dispersal tendency. In their simulations, low dispersal success of females resulted in a large number of solitary, unmated males, thus reducing population growth rate. Where one sex is more likely to disperse, or more likely to disperse a greater distance, biased sex ratios will occur in isolated populations, potentially impacting individual fecundity and population growth rate (Milner-Gulland et al., 2003; Gerber, 2006). We also do not know what motivates dispersal in Hawaiian gallinules, whether it is movement away from crowded sites or towards higher habitat quality (e.g., Doerr, Doerr & Jenkins, 2006; Pfluger & Balkenhol, 2014). Thus, our currently poor understanding of dispersal in Hawaiian gallinules would be improved not only by a general understanding of the frequency of between-wetland dispersal on O‘ahu, but also of sex-specific rates and mortality risk of dispersing individuals. Because sexes cannot be distinguished in the field, it will likely require assessments using genetic markers.

Several aspects of our vital rate estimates are worth careful examination. First, the vital rate estimates used in our baseline model come almost exclusively from managed populations (van Rees et al., 2018a), and these were applied to all subpopulations. However, we believe that these values are likely to be optimistic for unmanaged habitats, where habitat improvements (vegetation management and predator removal; VanderWerf, 2012; Underwood et al., 2013) are not occurring. The population projection models in this paper may thus fail to capture important differences in reproductive and survival parameters between subpopulations that would lead to reduced population sizes and persistence likelihoods. In addition, if the subspecies is delisted and management reduced, we anticipate extirpation risk to increase. Our vital rate data also came from a suite of short-term (1-5 year) studies spread across a 35-year time period (1979–2014), and accordingly may contain information from different phases of the Hawaiian gallinule’s population trajectory on O‘ahu over this time period (Reed et al., 2011), introducing variation that may not be typical of current conditions on the island. van Rees et al. (2018a) also stress that poor detection rates of Hawaiian gallinules likely caused us to underestimate longevity and mean annual adult survival. In using their less conservative estimate of survival, we have attempted to compensate for this potential bias.

Our population model included density-dependent feedbacks despite having only indirect evidence for their influence on gallinule populations. It has been shown that incorporating density dependence into population projection models reduces extinction risk by creating compensatory mechanisms that tend to return populations from declines (Ginzburg, Ferson & Akçakaya, 1990). We acknowledge that inclusion of this phenomenon may produce optimistic projections in our models.

Finally, an important consideration in our results is that our simulations assessed population viability only for gallinule populations on the island of O‘ahu, which represents roughly 50% of their range in area but possibly <30% of the entire statewide population (Hawaii biannual waterbird survey, 2016–2017, unpublished data).We did not expand the assessment to include Kaua’i because of our limited understanding of the vital rates, distribution, population connectivity, and abundance of Hawaiian gallinules on Kaua‘i. While the distribution of Hawaiian gallinules on O‘ahu is discrete and well-studied due to a long history of habitat loss and fragmentation on the island (van Rees & Reed, 2014; van Rees et al., 2018b), habitats and subpopulations are not as clearly delineated on Kaua‘i, and their connectivity and movement rates are unstudied. Applying the present model to a single-population scenario (lacking information on substructure) for Kaua‘i would be largely uninformative, given that the island-wide population is very large (in excess of 800 individuals; K Uyehara, USFWS, pers. comm., 2018) and that we would be unable to model the potential for subpopulation extirpation. In addition to having different vital rates from the O‘ahu populations (van Rees et al., 2018a), gallinules on Kaua‘i face a different suite of potential threats, being more at risk of flooding and with higher instances of botulism, but in the absence of high densities of mongoose *Herpestes auropunctatus* (Htun et al., 2016; van Rees et al., 2018a). These differences necessitate a separate population viability analysis for the island of Kaua‘i, which will not be possible until the distribution and connectivity of gallinule subpopulations on the island are better understood.

### Sea level rise

We found that a large portion of current gallinule habitat is expected to disappear due to sea-level rise. Based on our population model, however, this reduced carrying capacity is not expected to cause extirpation of the overall island population. There are reasons, however, to believe that our assessment is conservative. The ∼2m sea level rise scenario that we chose for our 160 year projection is probably a substantial underestimate of sea level by 2180 (∼160 years from present). Additionally, we did not take into account habitat degradation through salinization. Coastal freshwater wetlands on O‘ahu could be salinized either by storm surge or by saltwater intrusion in underlying basal aquifers (Kane et al., 2015), reducing their habitat value to gallinules. The impacts of salinization with sea level rise could thus be quite severe and drive reductions in carrying capacity well beyond the spatial extent modeled in our projections. Finally, our assumption that gallinule densities are uniform across habitats can be reasonably challenged. We have found that many of the areas most threatened by sea level rise are also those with the highest population densities of Hawaiian gallinules (van Rees & Reed, 2016–2017, unpublished data). For example, Hamakua Marsh (part of the Windward Complex) and James Campbell National Wildlife Refuge (part of the Kahuku Complex) account for 51% and 76% of the carrying capacities of their respective wetland complexes, and will be the first parts of their respective wetland complexes lost to sea level rise. The effects of climate change on gallinule carrying capacity, and therefore on extirpation risk, therefore, is likely to be greater than that estimated in this paper.

Wetland migration due to soil accretion is a potential mitigating effect against habitat loss for some coastal wetland types, in which wetland distributions shift inland and upland with rising sea levels (e.g., Traill et al., 2011). This phenomenon may have little benefit on O‘ahu, where most areas farther inland from wetlands are either densely developed or feature dramatically sloped topography that transitions directly from the coastal plain into mountains, leaving little room for wetland migration. Additionally, soil accretion takes place after inundation, and is thus of value primarily for salt water wetland habitats (Kirwan et al., 2016). The persistence of the O‘ahu’s two major strongholds for Hawaiian gallinules, the Windward and Kahuku complexes, may depend strongly on the migration of wetland habitats to higher elevations and increased management of wetland habitats within those regions that are less vulnerable to rising sea levels. Both Kawainui marsh in the Windward Complex and the Shrimp Farms in the Kahuku Complex are positioned inland and adjacent to high-density managed sites from which gallinules might easily emigrate under sea level rise. Management (which is currently limited at both sites) to increase carrying capacity at these higher-elevation sites could create habitat capable of supporting a large portion of the gallinules currently found in their respective wetland complexes. Similar inland wetland alternatives are not available for the Pearl Harbor Complex or Turtle Bay, meaning that more intensive and perhaps economically infeasible measures like habitat creation and land acquisition might be necessary to mitigate sea level rise impacts there.

The high population growth capacity of Hawaiian gallinules implies that management strategies that increase carrying capacity of existing wetlands that may be less threatened by climate change (on O‘ahu or other islands) may compensate for anticipated losses at sites with greater risk. Such restoration, (e.g., Kawainui marsh, US Army Corp of Engineers (USACE), 2008), and potential creation of new habitats would require a solid understanding of the habitat requirements of Hawaiian gallinules. Unfortunately, no quantitative data on habitat correlates of gallinule abundance or breeding success have been published, so a better understanding of determinants of habitat quality may be a research priority for this subspecies.

### Sensitivity analysis and management strategies

Both conventional and regression sensitivity analyses highlighted the importance of juvenile survival for population persistence in Hawaiian gallinules. This finding is especially relevant to management, given the general belief that exotic invasive predators have larger impacts on juvenile than adult mortality, and that predator management is one of the longest-implemented and logistically feasible management strategies for this subspecies (VanderWerf, 2012). Reproductive output and mean adult mortality were also shown to be important, and they are affected by the same management strategies. Consequently, the prevailing emphasis on predator control in current management plans for this subspecies should be considered highly justified in light of our population viability analysis. The high importance and uncertainty of our estimates of juvenile mortality make it a top priority for future field research on Hawaiian gallinules. Related to this, however, are unanswered questions pertaining to predator control to protect Hawaiian gallinules and other endemic waterbirds. Notably, which predators are having the largest impacts and at what life stage, how these effects differ across wetlands, islands, and years, and which management strategies are most effective at reducing the impacts of these predators. The management of endangered and introduced invasive species is constrained by finite budgets, so more applied research will be necessary to clarify the exact management implications of our results. It is additionally unclear if introduced predators are the main driver of juvenile mortality. Although this notion is supported by numerous anecdotal observations (C van Rees, pers. obs., 2018, A Nadig, USFWS, pers. comm., 2016) and prevailing beliefs (USFWS, 2011; VanderWerf, 2012) on O‘ahu, it is unsupported by empirical evidence. Accounts of emaciated chicks on Kaua‘i (K Uyehara, pers. obs., 2018) might imply that access to food also influences chick and juvenile survival rates. Understanding the mechanisms driving survival in these early life stages is essential to an effective management solution to low survival rates.

Our sensitivity analysis of mean brood size and the distribution of number of broods per year showed that the processes intrinsic to those parameters (nest success and hatch rates) have an important but secondary impact on the viability of Hawaiian gallinule populations on O’ahu. The importance of nest success and hatch rate for population viability is sensible, being that they represent a key stage in the gallinule’s reproductive cycle, but other idiosyncrasies of the gallinule’s reproductive biology may be reducing its importance. The readiness with which Hawaiian gallinules (and congeners) may limit the impact of individual nest failures (van Rees et al., 2018a), and a year-round nesting season indicates that multiple broods are easily possible in any given year. Accordingly, birds may continue attempting to breed until a nest is successful (as has been observed in one closely monitored pair, C van Rees, pers. obs., 2018), at which point juvenile survival becomes the final determinant of reproductive success.

The lower importance of dispersal rates on population growth rate and extirpation risk relative to juvenile and adult survival to population persistence in our study corresponds with findings in other systems and reinforces the notion that connectivity typically contributes less to population viability than demographic rates (South, 1999). In both regression-based and conventional sensitivity analyses, however, dispersal had some impact on the extirpation risk of small subpopulations, amounting to as much as half of the sensitivity value of directly altering carrying capacity for those populations. The extirpation of these subpopulations under low-dispersal scenarios caused a slow decline in overall island population throughout the study period, which was ameliorated by increasing the dispersal multiplier. Interestingly, dispersal multiplier values >10 caused smaller increases in mean island population size at 160 years, indicating that, at a certain point, gallinule emigration to isolated populations was detrimental to the overall population. This is similar to observations by Medici & Desbiez (2012), who examined metapopulation persistence in lowland tapirs (*Tapirus terrestris*). Our perturbation analysis also showed steady decreases in extirpation probability across a wide range of connectivity values. For small and especially medium populations, even modest (2–5×) increases over current dispersal rates can produce nontrivial changes in the probability of persistence of small Hawaiian gallinule subpopulations. These increases are within current variation in dispersal rates between populations, implying that they are within the realm of possibility for management. It is important to recognize that, according to our models, management actions that directly increase the carrying capacity of small subpopulations would be generally more effective at reducing extirpation risk. While creating new habitat for Hawaiian gallinules on the O’ahu might be prohibitive in social and economic cost due to constraints on land ownership and availability, the restoration of existing habitats would be a highly effective approach to increasing carrying capacities that would avoid such the complications of land acquisition. Increasing connectivity through dispersal might be a viable additional option for decreasing extirpation risk under future sea level rise conditions, where large subpopulations will be reduced to small and medium-sized ones, and more direct and effective methods of decreasing extirpation risk will become more challenging. Recent work by van Rees et al. suggests that stream networks and green water management infrastructure (e.g., drainage swales) proposed as solutions for climate change adaptation on O‘ahu may increase landscape permeability to gallinules, providing a feasible measure for managing connectivity for this subspecies.

## Conclusions

Our population model of the Hawaiian gallinules on O‘ahu predicts near-zero probability that the island’s population will be extirpated in the next 160 years under current conditions, but with the caveat that this persistence is primarily dependent on two to four large and medium subpopulations. The model predicted a high likelihood of extirpation for all smaller populations, which would lead to small overall population declines for the island as a decrease in genetic diversity. Sensitivity analysis highlighted juvenile survival as the most influential parameter affecting population growth rates and extirpation probabilities, implying that management methods (e.g., predator control) that decrease juvenile mortality rates may be the most effective approach to reducing extirpation risk in the population of this subspecies on O‘ahu. Our sea level rise projections reveal that it is a major future threat to Hawaiian gallinule populations, which we conservatively estimate could reduce the island’s current population by as much as 45%. Although dispersal was found to be largely unimportant to the persistence of the O‘ahu’s overall population, moderate increases in dispersal made nontrivial reductions in extirpation risk and population growth rate for small, isolated subpopulations. As sea level rise reduces the number and size of subpopulations on the island, the importance of dispersal for the subspecies’ overall persistence may increase relative to other conservation measures.

##  Supplemental Information

10.7717/peerj.4990/supp-1 Supplemental Information 1 Count data of Hawaiian gallinules from playback surveys during Summer 2017 Each line represents the results of counts taken using the playback method of DesRochers, Gee & Reed (2008) at a particular pond in each wetland complex. Totals for individual wetlands and individual wetland complexes are presented. Each count was carried out using 1 minute of playback followed by 1 minute of silence, recording all adult birds seen during that period; these were repeated at 20 m intervals around the entire perimeter of each pond.

10.7717/peerj.4990/supp-2 Figure S1 Spatial representation of potential Hawaiian gallinule habitat loss due to sea level rise over 80 and 160 year time scales at the Windward wetland complex on O’ahu Light gray areas indicate current Hawaiian gallinule habitat, dark gray indicates seawater. Black areas indicate habitat lost due to seawater inundation. ∼6% of habitat in the Windward complex is lost with ∼1 m sea level rise (modeled here as 80 years), and >54%, under ∼2 m sea level rise (modeled here as 160 years).

10.7717/peerj.4990/supp-3 Figure S2 Spatial representation of potential Hawaiian gallinule habitat loss due to sea level rise over 80 and 160 year time scales at the Pearl Harbor wetland complex on O’ahu Light gray areas indicate current Hawaiian gallinule habitat, dark gray indicates seawater. Black areas indicate habitat lost due to seawater inundation. ∼‘0% of habitat in the Pearl Harbor Complex is lost with ∼1 m sea level rise (modeled here as 80 years), and >99% under ∼2 m sea level rise (modeled here as 160 years).

10.7717/peerj.4990/supp-4 Figure S3 Spatial representation of potential Hawaiian gallinule habitat loss due to sea level rise over 80 and 160 year time scales at Turtle Bay resorts on O’ahu. 160 years) Light gray areas indicate current Hawaiian gallinule habitat, dark gray indicates seawater. Black areas indicate habitat lost due to seawater inundation. No habitat in Turtle Bay is lost with 1m sea level rise (modeled here as 80 years), and >99% is lost under ∼2 m sea level rise (modeled here as 160 years).

10.7717/peerj.4990/supp-5 Figure S4 Perturbation analysis examining the sensitivity of extirpation probability among Hawaiian gallinule populations on O’ahu to varying proportions of females in the breeding pool Points represent parameter values tested. Results are shown for the overall population (top) and 5 subpopulations.

10.7717/peerj.4990/supp-6 Figure S5 Perturbation analysis examining the sensitivity of extirpation probability among Hawaiian gallinule populations on O’ahu to mean brood size Points indicate parameter values tested. Results are shown for the overall population (top) and 5 subpopulations.

10.7717/peerj.4990/supp-7 Supplemental Information 2 Raw data and code for Hawaiian gallinule PVA A zipped folder containing raw data, Excel spreadsheet calculations, R code, and Vortex outputs used in preparing input data and analyzing outputs of Hawaiian gallinule population viability model implemented in Vortex.

## Additional Information and Declarations

### Data Availability

The following information was supplied regarding data availability: The raw data and code are provided in a Supplemental File.
