# Supplementary figures and images for: Predicted effects of landscape change, sea level rise, and habitat management on the extirpation risk of the Hawaiian common gallinule (Gallinula galeata sandvicensis) on the island of O‘ahu
Source: PeerJ. 2018 Jun 22;6:e4990. doi: 10.7717/peerj.4990 (PMC6016525; doi:10.7717/peerj.4990)

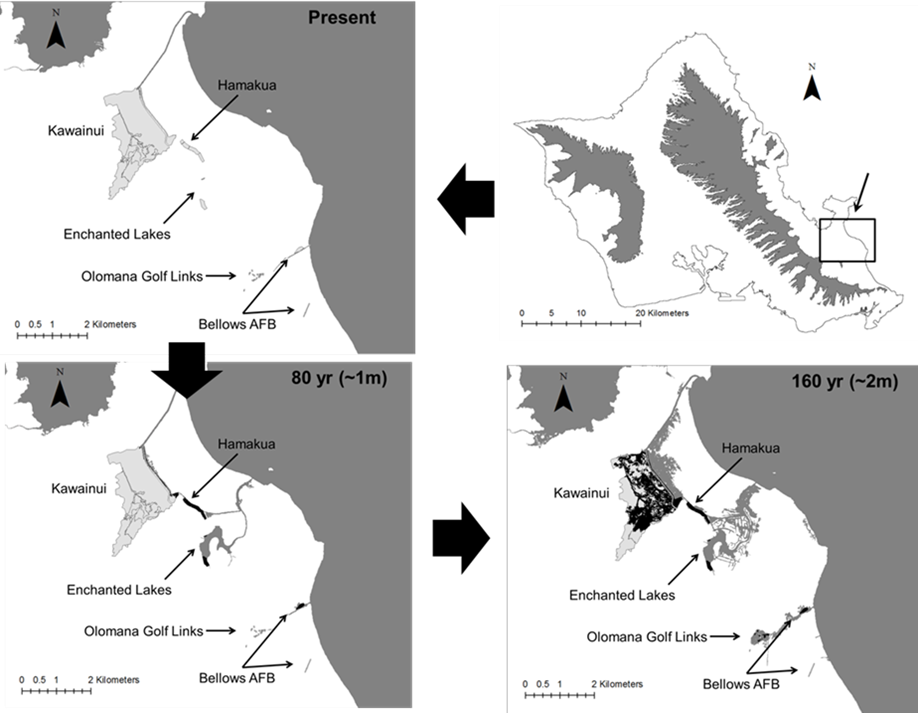

Supplement: Figure S1 — Light gray areas indicate current Hawaiian gallinule habitat, dark gray indicates seawater. Black areas indicate habitat lost due to seawater inundation. ∼6% of habitat in the Windward complex is lost with ∼1 m sea level rise (modeled here as 80 years), and >54%, under ∼2 m sea level rise (modeled here as 160 years). [file peerj-06-4990-s002.png]

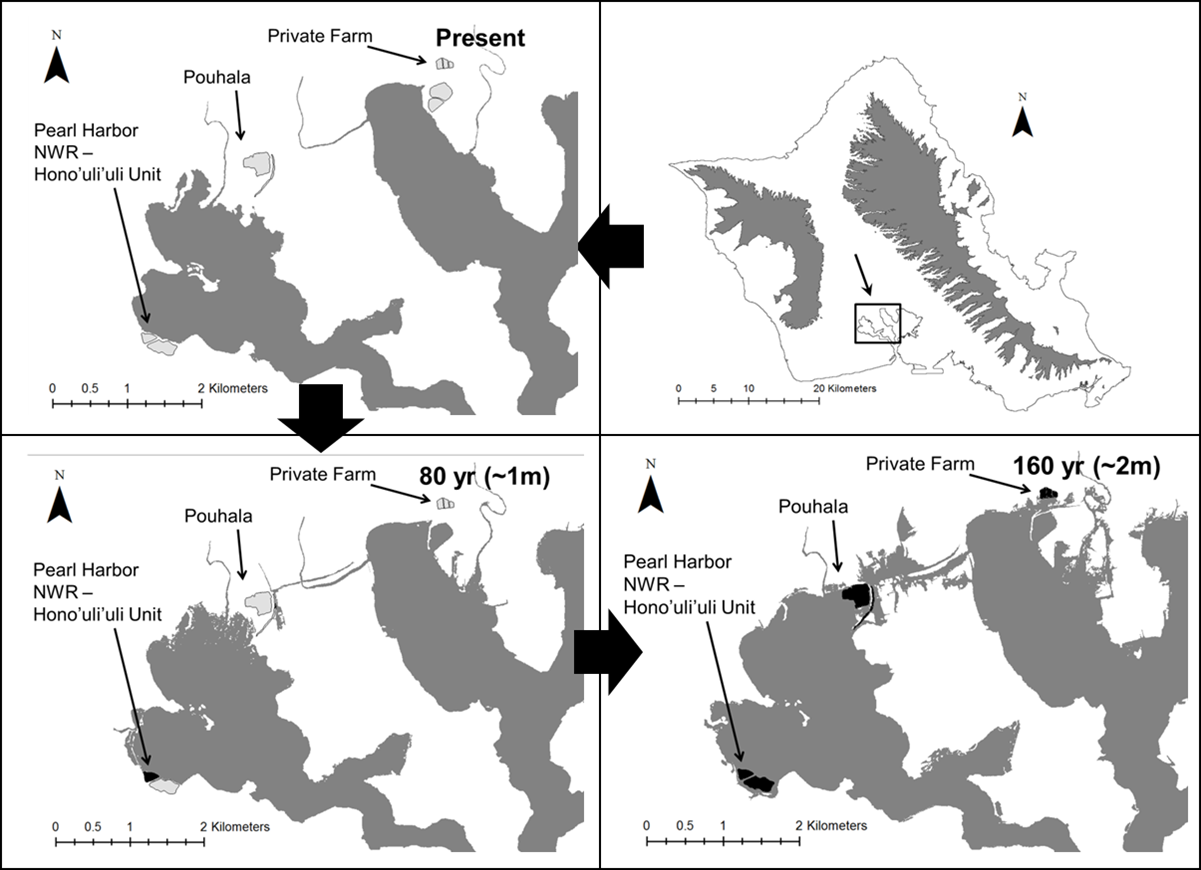

Supplement: Figure S2 — Light gray areas indicate current Hawaiian gallinule habitat, dark gray indicates seawater. Black areas indicate habitat lost due to seawater inundation. ∼‘0% of habitat in the Pearl Harbor Complex is lost with ∼1 m sea level rise (modeled here as 80 years), and >99% under ∼2 m sea level rise (modeled here as 160 years). [file peerj-06-4990-s003.png]

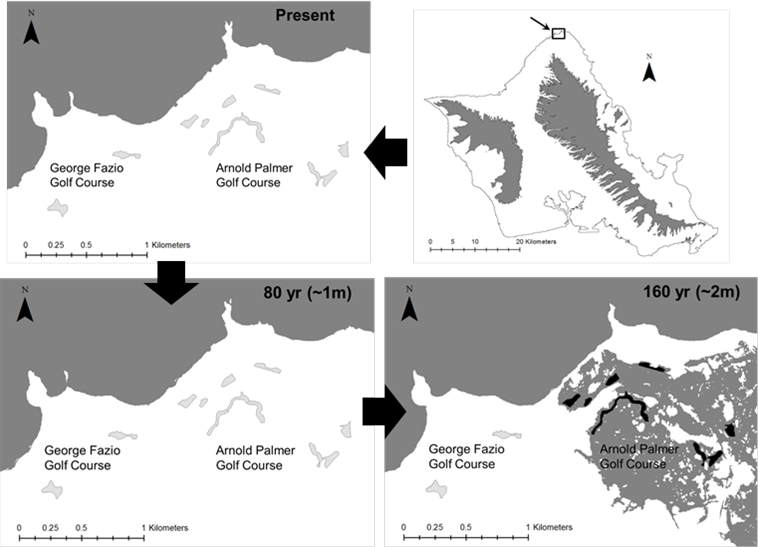

Supplement: Figure S3 — Light gray areas indicate current Hawaiian gallinule habitat, dark gray indicates seawater. Black areas indicate habitat lost due to seawater inundation. No habitat in Turtle Bay is lost with 1m sea level rise (modeled here as 80 years), and >99% is lost under ∼2 m sea level rise (modeled here as 160 years). [file peerj-06-4990-s004.png]

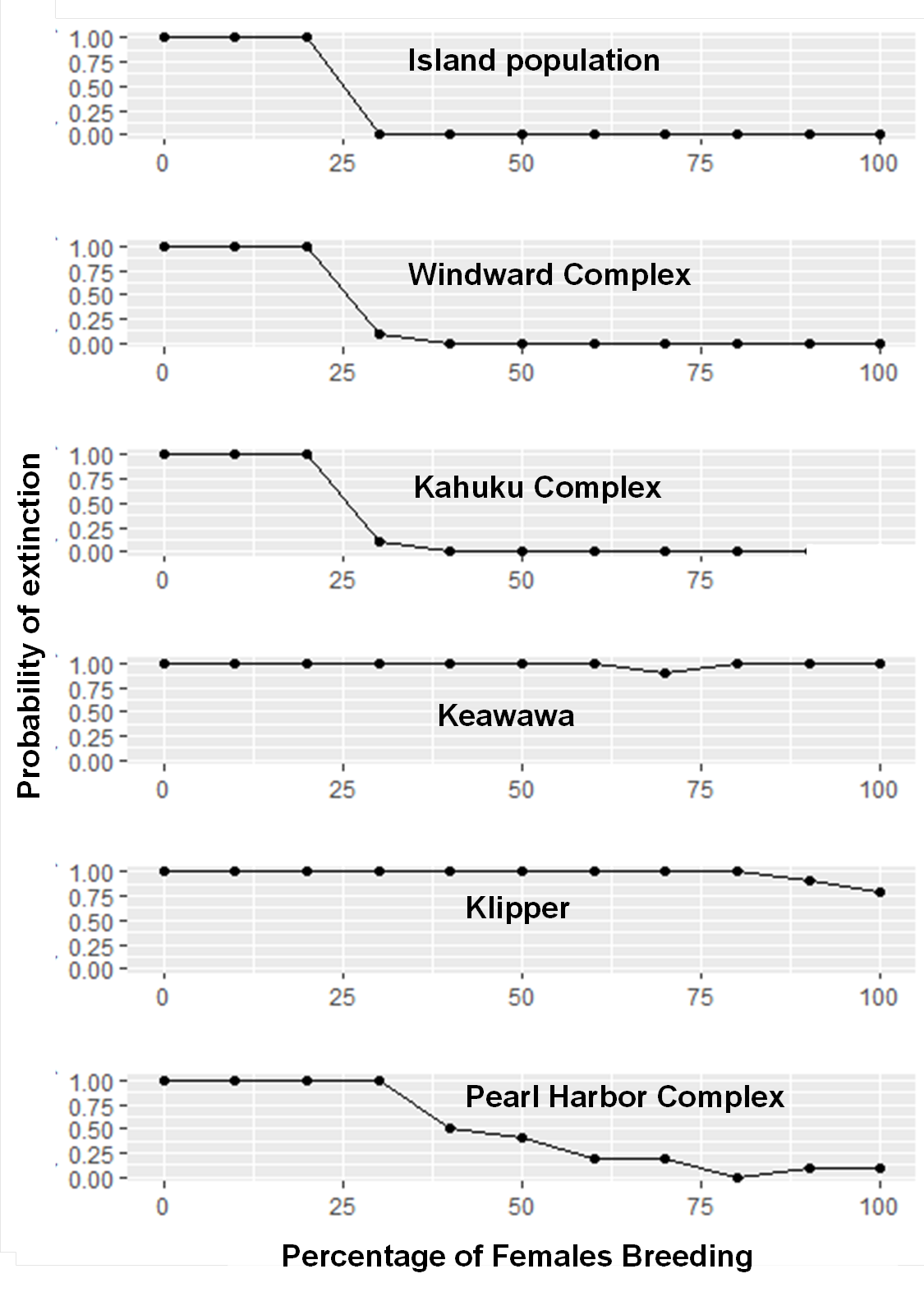

Supplement: Figure S4 — Points represent parameter values tested. Results are shown for the overall population (top) and 5 subpopulations. [file peerj-06-4990-s005.png]

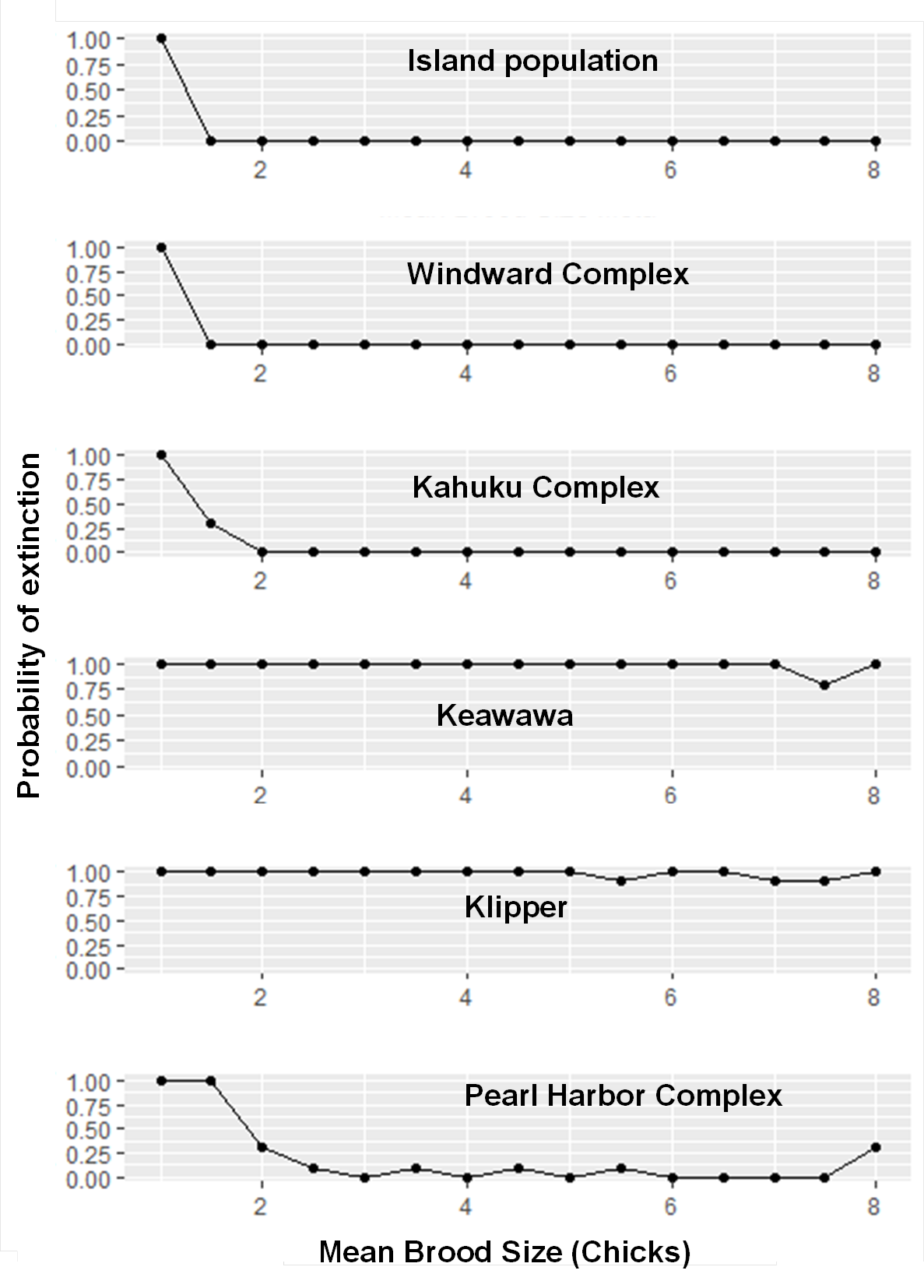

Supplement: Figure S5 — Points indicate parameter values tested. Results are shown for the overall population (top) and 5 subpopulations. [file peerj-06-4990-s006.png]
